# A Case of Allergic Urticaria After Ophthalmic Nepafenac Use

**DOI:** 10.4274/tjo.78614

**Published:** 2018-06-28

**Authors:** Erdoğan Yaşar, Deniz Öztürk Kara, Nilgün Yıldırım

**Affiliations:** 1 Aksaray University Aksaray Training and Research Hospital, Ophthalmology Clinic, Aksaray, Turkey; 2 Aksaray University Aksaray Training and Research Hospital, Dermatology Clinic, Aksaray, Turkey; 3 Osmangazi University Faculty of Medicine, Department of Ophthalmology, Eskişehir, Turkey

**Keywords:** Nepafenac, allergic, urticaria

## Abstract

A 21-year-old male patient with no history of systemic disease or drug use presented to our clinic with redness and pain in the right eye. Best corrected visual acuity was 20/20 in both eyes. Inflamed pinguecula was observed on slit-lamp examination and the patient was prescribed ophthalmic nepafenac eye drops. After instilling the drops that day and the next day, the patient presented again due to pruritus and rash. Upon consultation with the dermatology department, the patient was diagnosed with drug-induced allergic urticaria and the nepafenac drops were discontinued. Although urticaria has been reported as a side effect after systemic non-steroidal anti-inflammatory drug (NSAID) use, such a reaction has not been reported with an ophthalmic NSAID and ours is the first reported case of urticaria following ophthalmic nepafenac use. This unique case highlights the fact that ophthalmologists must also keep urticaria in mind as a potential side effect when prescribing this drug.

## Introduction

Non-steroidal anti-inflammatory drugs (NSAIDs) are commonly used in oral, intramuscular and topical (skin and ophthalmic) forms for a variety of indications. Ophthalmic NSAIDs currently in use include nepafenac, ketorolac tromethamine, diclofenac sodium, bromfenac and flurbiprofen. A study performed in rabbit eyes demonstrated the distribution of ophthalmic nepafenac in the cornea, aqueous humor, iris, ciliary body and choroid.^1^ These ophthalmic drugs are used in the management of inflammatory ocular diseases, allergic conjunctivitis and postoperative pain following refractive and cataract surgery and in the treatment of cystoid macular edema after cataract surgery.^2,3,4,5,6^

Adverse effects of ophthalmic NSAIDs include corneal melting,^7,8,9,10^ ocular tissue hemorrhage,^7^ blurred vision, photophobia, posterior capsule opacity, foreign body sensation, dry eye and increased intraocular pressure.^11^ Adverse effects involving the pulmonary, gastrointestinal, dermatologic, renal, cardiovascular, hematologic, pulmonary and central nervous systems have been reported after topical, intramuscular and oral administration.^12,13,14,15,16,17,18,19^ Here we present urticaria as a previously unreported adverse effect of an ophthalmic NSAID.

## Case Report

**Figure 2:**
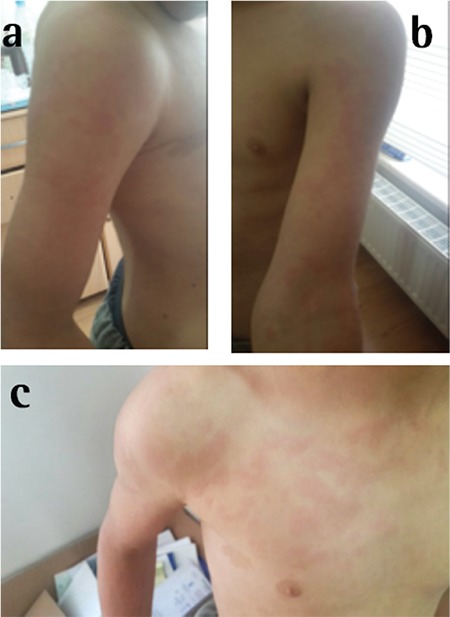
Erythematous and edematous plaques on the patient’s right arm (a), left arm (b), and upper trunk (c)

A 21-year-old male patient presented to our clinic with pain and redness in his right eye. On physical examination, visual acuity using Snellen chart was 20/20 in both eyes and intraocular pressures were 14 and 15 mmHg in the right and left eyes, respectively. On slit-lamp biomicroscopic examination, minimally inflamed pinguecula was noted on the nasal conjunctiva of the right eye. No pathology was observed in the left eye except pinguecula (Figure 1a, b). Fundus examination revealed no pathology in either eye. The patient reported no disease or drug use in his systemic medical history. Treatment was initiated with ophthalmic nepafenac (Nevanac 0.1%, Alcon) four times daily and the patient was scheduled for follow-up one week later. The next day, the patient returned to the outpatient clinic due to redness and itching on his body. He stated that an itchy rash had formed on his trunk and arms the previous day, approximately 1-2 hours after instilling the nepafenac eye drop and he had been treated for allergy that night in the emergency department. A similar reaction had occurred 1-2 hours after instilling the drop that morning and the dermatology department was consulted. Erythematous, edematous plaque lesions were observed on the arms, neck and abdomen on dermatologic examination and the patient was diagnosed with allergic urticaria by the dermatologist (Figure 2a, b, c). The dermatologist instructed the patient to discontinue the nepafenac drops and prescribed oral antihistamines to treat the urticaria. The ophthalmology department recommended preservative-free lubricating drops and scheduled the patient for follow-up. At follow-up three days later, the patient’s skin lesions and symptoms had completely regressed and his ocular complaints had also improved.

## Discussion

NSAIDs act by inhibiting the enzyme cyclooxygenase (COX), thus reducing the synthesis of prostaglandin, prostacyclin and leukotriene from arachidonic acid. There are two forms of COX. COX-1 is generally found in all tissues and plays a protective role by regulating the action of prostaglandins. COX-2 increases inflammation by stimulating immune system cells and other tissues in the presence of various stimuli such as mitogens, inflammatory cytokines and tumor promoters.^20^

Ophthalmic NSAIDs currently in use include nepafenac, ketorolac tromethamine, diclofenac sodium, bromfenac and flurbiprofen. The chemical designation of nepafenac is 2-amino-3-benzoylbenzeneacetamine and it is available as a 0.1% suspension. Ophthalmic nepafenac is the only prodrug among the NSAIDs. It is deaminated to form amfenac, a potent COX inhibitor. Ophthalmic nepafenac targets the anterior segment and intraocular vascular tissues. An *in vivo* study in humans showed nepafenac had a significantly shorter time to peak anterior chamber concentration after instillation on the cornea, followed by amfenac, ketorolac and bromfenac.^21^ Ophthalmic nepafenac takes effect approximately 15 minutes after topical application and lasts more than 8 hours.^22^ Quantitative plasma concentrations of nepafenac and amfenac were measured in subjects 2-3 hours after ocular administration and mean steady-state C-max values of the drugs were 0.310±0.104 ng/mL and 0.422±0.121 ng/mL, respectively. Ophthalmic diclofenac has been associated with corneal melting in studies of the ophthalmic side effects of topical NSAIDs.^7^ In another study, topical ketorolac and bromfenac were associated with severe corneal damage and the authors suggested that patients with corneal damage should be asked about their use of these agents.^8,9^ Topical nepafenac has also been associated with corneal melting.^10^ Ophthalmic NSAIDs may prolong bleeding time by impairing platelet aggregation, thus leading to hemorrhage in ocular tissues.^7^ Therefore, caution is warranted when using ophthalmic NSAIDs long-term in patients using systemic NSAIDs, patients who smoke or use alcohol and in elderly and pediatric populations. In a study of the ocular side effects of nepafenac, ocular adverse events that occurred at rates of at least 1% included blurred vision, photophobia, posterior capsular opacity, foreign body sensation, dry eye and increased intraocular pressure.^11^

Adverse effects have also been reported after using topical and intramuscular NSAIDs. In one of these reports, a patient with asthma history experienced an asthma attack after using piroxicam topical gel (NSAID) for knee pain.^15^ Another patient with no history of gastric ulcer developed gastric ulcer perforation four days after starting intramuscular ketorolac (NSAID) treatment for traumatic humerus and femur fracture.^16^ The systemic side effects of oral NSAIDs on the gastrointestinal, renal, cardiovascular, hematological, pulmonary and central nervous systems have been demonstrated in various studies.^17,18,19,20^ Dermatologic side effects include urticaria, morbilliform and vesiculobullous eruptions, exfoliative erythroderma, erythema multiforme, Steven Johnson syndrome and toxic epidermal necrosis.^21,22^Urticaria occurs as the result of mediator release from mast cells or basophils after contact with a triggering stimulus. These mediators induce vasodilation and transudation from small vessels, which causes the development of the characteristic erythematous, edematous, itchy papules and plaques. Many factors are implicated in the etiology of urticaria. The main etiological causes of acute urticaria are drugs, food and infections. It is usually possible to determine the etiology based on only a detailed history. Nearly all drugs can cause urticaria but the most common are antimicrobials (penicillin, sulfonamides), analgesics and antiinflammatory drugs (acetylsalicylic acid, NSAIDs, opiates), angiotensin converting enzyme (ACE) inhibitors and blood products.^23,24^ In a study conducted in rabbits, it was determined that ocular instillation of 0.5% (50 µL) diclofenac resulted in a peak plasma concentration of 185 ng/mL after 15 minutes at the earliest.^25^ In addition, it has been shown in rabbits that 7-10% of ophthalmic flurbiprofen enters the ocular circulation, while 74% passed to the systemic circulation.^26^ Urticaria is a known adverse effect of systemic NSAID use and we believe that our patient developed it after the ophthalmic NSAID entered the systemic circulation via the conjunctival vessels and nasolacrimal duct. Although there are previous reports of allergic urticaria after oral NSAID use,^23,24^ our case is novel as the first reported case in the literature of allergic urticaria as an adverse event after ophthalmic NSAID use.

## Figures and Tables

**Figure 1a:**
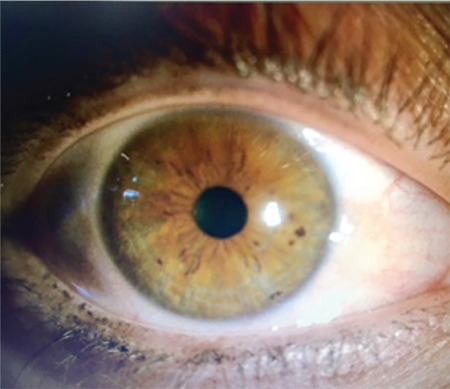
Inflamed pinguecula in the right eye

**Figure 1b:**
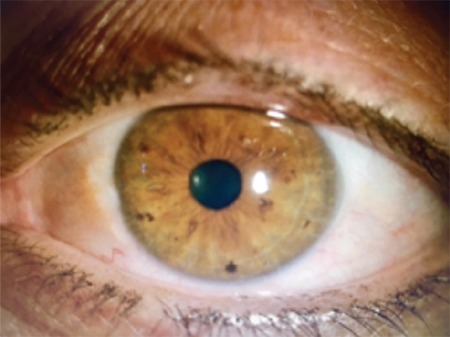
Pinguecula in the left eye
